# Predicting Subcontractor Performance Using Web-Based Evolutionary Fuzzy Neural Networks

**DOI:** 10.1155/2013/729525

**Published:** 2013-06-19

**Authors:** Chien-Ho Ko

**Affiliations:** Department of Civil Engineering, National Pingtung University of Science and Technology, 1 Shuefu Road, Neipu, Pingtung 912, Taiwan

## Abstract

Subcontractor performance directly affects project success. The use of inappropriate subcontractors may result in individual work delays, cost overruns, and quality defects throughout the project. This study develops web-based Evolutionary Fuzzy Neural Networks (EFNNs) to predict subcontractor performance. EFNNs are a fusion of Genetic Algorithms (GAs), Fuzzy Logic (FL), and Neural Networks (NNs). FL is primarily used to mimic high level of decision-making processes and deal with uncertainty in the construction industry. NNs are used to identify the association between previous performance and future status when predicting subcontractor performance. GAs are optimizing parameters required in FL and NNs. EFNNs encode FL and NNs using floating numbers to shorten the length of a string. A multi-cut-point crossover operator is used to explore the parameter and retain solution legality. Finally, the applicability of the proposed EFNNs is validated using real subcontractors. The EFNNs are evolved using 22 historical patterns and tested using 12 unseen cases. Application results show that the proposed EFNNs surpass FL and NNs in predicting subcontractor performance. The proposed approach improves prediction accuracy and reduces the effort required to predict subcontractor performance, providing field operators with web-based remote access to a reliable, scientific prediction mechanism.

## 1. Introduction

A construction project involves various work items that need to be accomplished by subcontractors, including earthwork, formwork, concrete pouring, plastering, rebar, and mechanical and electrical tasks. Subcontractor performance directly influences project cost, duration, quality, and safety [1–3]. Project success cannot be achieved without appropriate performance on the part of the subcontractors [4]. The general contractor's key responsibility is selecting subcontractors with the capacity to perform the required work [5–7]. When selecting a subcontractor, general contractors frequently use the subcontractor's previous performance as a reference for their future outcome [8]. However, this approach leaves much to be desired and general contractors could benefit significantly from techniques which would allow greater accuracy in predicting subcontractor future performance [9].

Many studies have been devoted to enhancing the performance assessment of construction subcontractors. Ekström et al. [10] used source credibility theory to assess subcontractor performance in architecture/engineering/construction (AEC) using a weighted rating tool. Mbachu [11] investigated the key criteria for assessing subcontractor performance at the construction stage. Their research found that a subcontractor's previous performance is the most critical criterion for selecting high-performing subcontractors at the prequalification stage and for assessing their performance at the construction stage. Lean construction, a relatively new research area in the construction industry, has also been used to enhance subcontractor performance assessment [12]. Maturana et al. [13] conducted weekly assessments of subcontractor performance, rating quality, schedule fulfillment, safety, and cleanliness in terms of “good,” “regular,” or “bad.” Evaluation results were fed back to the general contractor for continuous improvement based on lean principles. 

While most studies have focused on enhancing subcontractor performance assessment, a few investigations have investigated methods of predicting subcontractor performance. Le-Hoai et al. [14] applied multiple regression analysis techniques to integrate significant variables including subcontractor selection to predict project length. Park [15] investigated critical success factors for whole life performance assessment, placing the identified factors into a criteria matrix to aid decision making for selecting subcontractors at the bid stage. Another investigation conducted by Elazouni and Metwally [16] developed a decision support system that assigns work items to subcontractors under constraints and predicts the project's final profit. Previous studies have considered subcontracting and subcontractor performance as factors for project success, but construction projects involve a variety of subcontractors. A successful outcome relies directly on the aggregated success of these subcontractors. One way to achieve project success is to predict subcontractor performance and base subcontractor selection on their predicted ability to adequately perform the work. 

The process of predicting subcontractor performance is complex, full of uncertainty, and highly contextualized, and it thus relies on decisions by experts [17]. Artificial intelligence (AI) is concerned with building computer systems that solve problems intelligently by emulating human behavior [18], making AI suitable for predicting subcontractor performance. The most popular AI paradigms are genetic algorithms (GAs), fuzzy logic (FL), and neural networks (NNs) [19]. These three techniques simulate different aspects of biological behaviors. The GA is a stochastic searching process based on natural selection and natural genetics [20]; FL simulates high level human decision-making processes [21]; NNs model brain functions [22]. Each method offers certain benefits for problem solving, and combining GAs, FL, and NNs provides potentially combines these benefits to provide a promising direction for predicting subcontractor performance.

The objective of this research is to develop Evolutionary Fuzzy Neural Networks (EFNNs) to predict subcontractor performance. In EFNNs, a floating point codification is used to encode parameters required in Fuzzy Neural Networks (FNNs). A multi-cut-point crossover is adopted to explore the parameters required in NNs and FL. To improve implementation convenience, a web-based system is developed to facilitate decision-making processes. 

This research first introduces practices used to predict subcontractor performance in the construction industry.  Section 3 explains the development of the EFNNs, with a detailed discussion of evolutionary processes, floating number codifications, and the multi-cut-point crossover operator. Applicability of the EFNNs is validated in Section 4, comparing the performance of EFNNs, FL, and NNs in real cases. Finally, the paper concludes with suggestions for future research directions.

## 2. Performance Prediction Practice

Historical performance serves as an important indicator for general contractors use in selecting subcontractors [23]. Predicting subcontractor performance can be treated as a process in which previous patterns are applied to the present condition. In this situation, the mapping between the previous behavior and later performance is unknown. In addition, subcontractor performance is affected by various knowable and unknowable factors, such as management ability, site working condition, and subjective assessment [24, 25]. Thus, predicting subcontractor performance is a complex process based on uncertain information, thus requiring the knowledge and experience of experts. Current practice predicts subcontractor performance through the subjective perception of the manager. Diverse backgrounds and work experience may result in significant prediction discrepancies. AI techniques, which involve machine learning and optimization to mimic human decision-making process, may provide a scientific approach to overcome these drawbacks.

## 3. Evolutionary Fuzzy Neural Networks

### 3.1. Architecture

**Figure 1:**
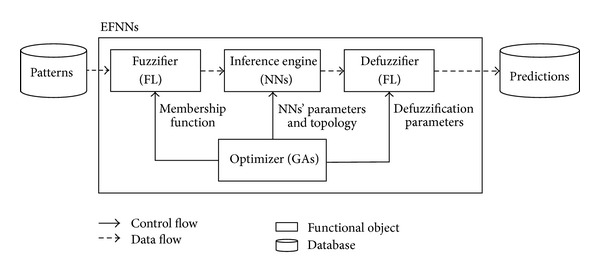
Architecture of Evolutionary Fuzzy Neural Networks.

Figure 1 shows the EFNNs architecture as a synergism of GAs, FL, and NNs. In EFNNs, NNs are used to learn the complex association between a subcontractor's previous performance and future status from historical data; FL is used to simulate high-level managerial decision making processes; GAs are used to achieve the optimal parameters required in NNs and FL, including distributions of the membership function, NN topology, and defuzzification parameters. Prediction results are stored in the database.

### 3.2. Adaptation Process

**Figure 2:**
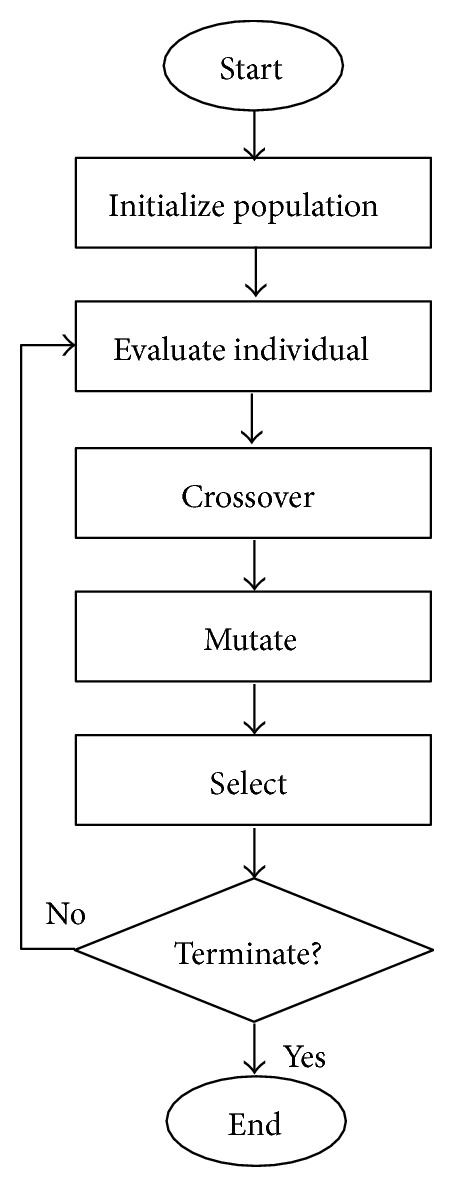
Evolutionary process of Evolutionary Fuzzy Neural Networks.

EFNNs optimize the required parameters using GAs.  Figure 2 displays the evolution process, which is explained next.

#### 3.2.1. Initializing Population

The EFNNs adaptation process first randomly generates a set of initial solutions. Each solution encodes variables into a floating Fuzzy Neural Network (FNN) string to simulate a natural chromosome. Every FNN string comprises of two segments: an MF substring and an NN substring.

**Figure 3:**
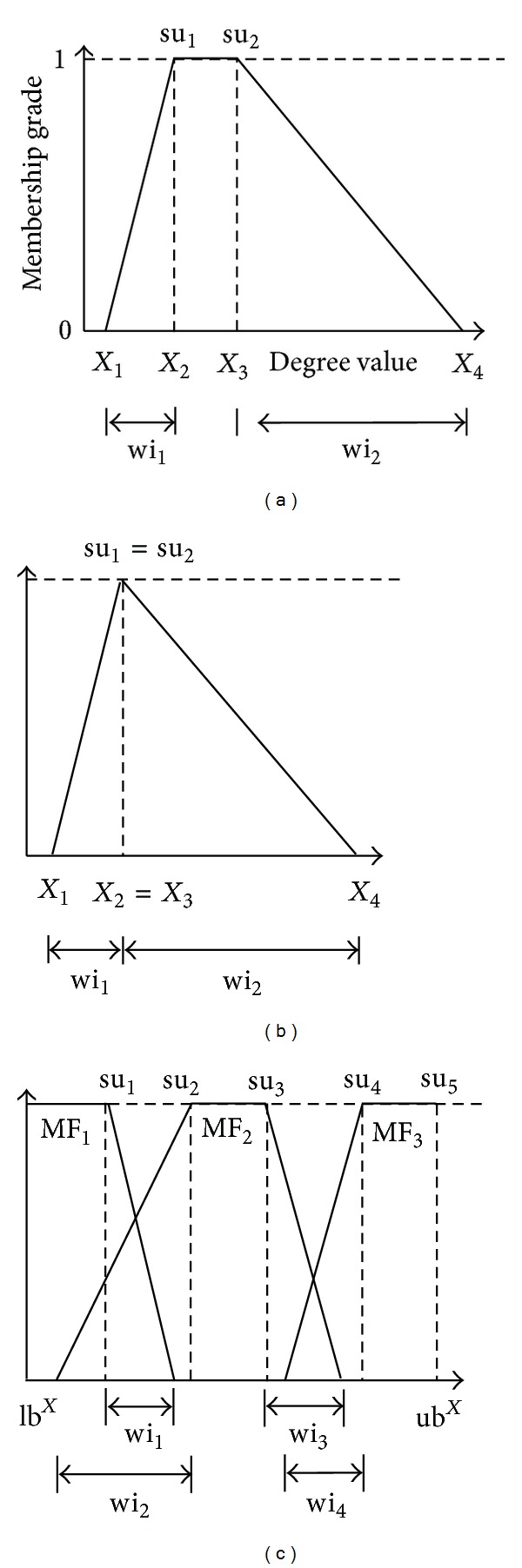
Membership functions: (a) trapezoidal MF; (b) special case of trapezoidal MF; (c) complete MF Set.

**Figure 4:**
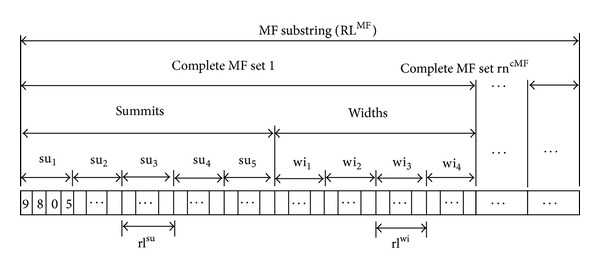
Summit and Width Representation Method (SWRM).

*MF Substring. *A Summit and Width Representation Method (SWRM) method [26] is used to encode membership functions (MFs) using floating numbers. The SWRM defines the distributions of uneven MFs by its summits and widths as shown in Figure 3. In Figure 3(a), the summits of the MF are su_1_ and su_2_ while the left and right widths are wi_1_ and wi_2_. A triangular MF can be regarded as a special case of a trapezoidal MF when su_1_ = su_2_ (see Figure 3(b)). For modeling problems using either trapezoidal MFs or triangular MFs, a complete MF set includes two shoulder MFs (see Figure 3(c)). The complete MF set shown in Figure 3(c) can thus be encoded using the SWRM, as demonstrated in Figure 4.

Using the SWRM, the required length of the floating numbers of MF substring RL^MF^ for encoding MFs is carried out as follows:

(1) $$\begin{array}{c} \text{R}{\text{L}}^{\text{MF}}=\text{r}{\text{n}}^{\text{cMF}}\times \left(n^{\text{su}}\times \text{r}{\text{l}}^{\text{su}}+n^{\text{wi}}\times \text{r}{\text{l}}^{\text{wi}}\right), \end{array}$$

where rn^cMF^ is the required number of the complete MF sets, *n* ^su^ is the number of summits in a complete MF set, rl^su^ is the required length for a summit depending on the demand, *n* ^wi^ is the number of widths in one complete MF set, and rl^wi^ is the required length for a width depending on the demand. 

The mapping from a domain [lb^*x*^, ub^*x*^] to a required length rl^*x*^ for variable *x* can be written as

(2) $$\begin{array}{c} 10^{\text{r}{\text{l}}^{x}-1}<\left(\text{u}{\text{b}}^{x}-\text{l}{\text{b}}^{x}\right)\times 10^{\text{rp}}\leq 10^{\text{r}{\text{l}}^{x}}-1, \end{array}$$

where rp is the required number of places after the decimal point and lb^*x*^ and ub^*x*^ are the lower and upper bound values of the variable *x*. Taking log functions on both sides of previous right-hand parts yields

(3) $$\begin{array}{c} \text{r}{\text{l}}^{x}=\left\lceil \frac{\log\left(\left(\text{u}{\text{b}}^{x}-\text{l}{\text{b}}^{x}\right)\times 10^{\text{rp}}+1\right)}{\log\left(10\right)}\right\rceil . \end{array}$$

The length of the floating numbers required for variables displayed in Figure 4 can be calculated using (3).

**Figure 5:**
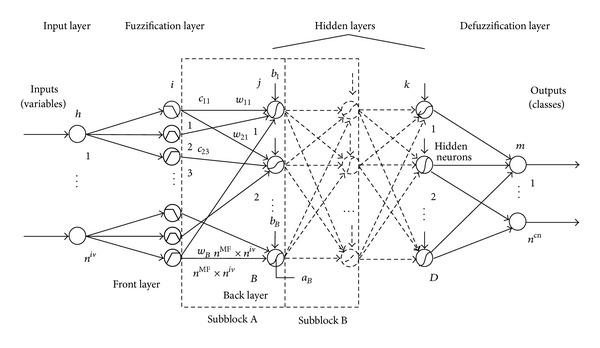
Neural networks structure.

*NN Substring*. A Block Representation Method (BRM) [26] is used to represent the NN floating numbers. The BRM describes the NN by its topology and network parameters (see Figure 5). The NN topology consists of the input layer and its neurons, the fuzzification layer and its neurons, many hidden layers and their hidden neurons, and the defuzzification layer and its neurons. The NN parameters include interconnections, weight values, bias values, and the slopes of the activation functions.

**Figure 6:**
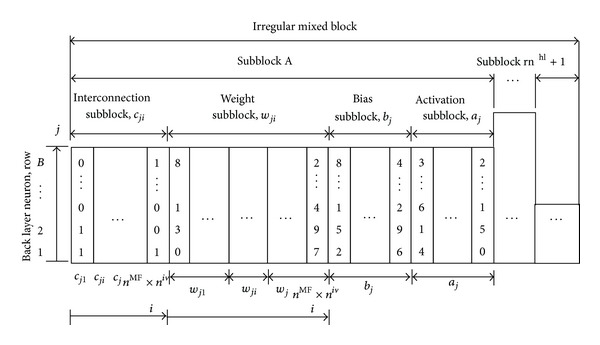
Block Representation Method (BRM).

The number of hidden layers and their hidden neurons of the NN are randomly generated using the BRM. A random number of hidden layers rn^hl^ is generated in [lb^hl^, ub^hl^] where lb^hl^ and ub^hl^ are the lower and upper bounds of the hidden layers. The method then generates rn^hl^ random numbers (rn^hn^) to determine the hidden neurons of each hidden layer. Each random number rn^hn^ is generated between lb^hn^ and ub^hn^ where lb^hn^ and ub^hn^ denote the lower and upper bounds of the hidden neurons. According to the generated topologies, the BRM calculates the required spaces to represent the NN. The method for encoding NNs is shown in Figure 6.

In Figure 6, the “Subblock A” represents the relationship between the fuzzification layer (front layer) and the first hidden layer (back layer). The height of the subblock (*h*_A_^sb^) directly indicates the number of hidden neurons in the back layer. Each row of the “Subblock A” represents one neuron of the back layer. The length of the NN substring, *L* ^NN^, is expressed as

(4) $$\begin{array}{c} L^{\text{NN}}=\sum_{i=\text{Sub-block}\;\text{A}}^{\text{Sub-block}(\text{r}{\text{n}}^{\text{hl}}+1)}\left(h_{i}^{\text{sb}}\times {\text{rcn}}_{i}^{\text{sb}}\right), \end{array}$$

where *h*_*i*_^sb^ is the height of subblock *i* and rcn_*i*_^sb^ is the required width of subblock *i*. The required column number (width) of subblock *i* (noted with rcn_*i*_^sb^) can be calculated using (5). The length of the floating numbers for the variables can be calculated using (3). Consider

(5) $$\begin{array}{c} {\text{rnc}}_{i}^{\text{sb}}={\text{nn}}_{i}^{\text{fl}}\times \left({\text{rcn}}_{i}^{\text{in}}+{\text{rcn}}_{i}^{\text{we}}\right)+{\text{rcn}}_{i}^{\text{bi}}+{\text{rcn}}_{i}^{\text{as}}. \end{array}$$

The MF substring encodes the distribution of MFs, and the NN substring encodes the NN parameters. To find the optimum combination of MFs and NNs, the MF substring and NN substring are combined. A complete chromosome, an FNN string *L* ^FNN^, is defined by (6). Via evolutionary processes, the combined chromosome concurrently identifies the optimum decision variables, where

(6) $$\begin{array}{c} L^{\text{FNN}}=\text{R}{\text{L}}^{\text{MF}}+L^{\text{NN}}. \end{array}$$

#### 3.2.2. Evaluating Individual Chromosomes

The adaptation process is designed to obtain EFNNs with high accuracy and good generalization properties. The EFNNs accuracy on input patterns can be improved by increasing network complexity. However, an accurate fit of the network to the input patterns does not mean that the overall problem behaviors are captured well [27]. A large network size also entails a higher computational cost and generally suffers from overfitting of data in input patterns and deterioration of generalization properties [28]. Thus, the objective of the adaptation process is to preserve acceptable levels of prediction accuracy using the fittest shapes of MFs with the minimum NN topology and optimum NN parameters. This is posed as an optimization problem. The objective function of the EFNNs is a combination of prediction accuracy and network complexity as follows:

(7) $$\begin{array}{c} v^{\text{ob}}=c^{\text{aw}}\times s^{\text{er}}+c^{\text{cw}}\times \text{mc}, \end{array}$$

where *v* ^ob^ is the objective value, *c* ^aw^ is the accuracy weighting coefficient, *s* ^er^ is the error signal, *c* ^cw^ is the complexity weighting coefficient, and mc is the network complexity. 

#### 3.2.3. Crossover

**Figure 7:**
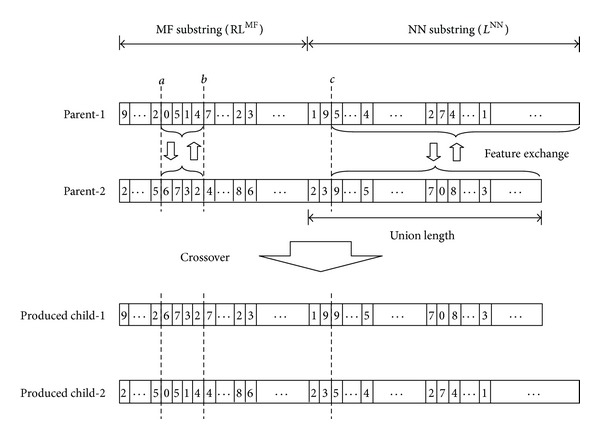
Three-cut-points crossover.

The crossover repeatedly exchanges high performance notations in attempting to improve performance. It operates on a pair of parent chromosomes and produces two children by exchanging the parent features. EFNNs use a three-cut-point crossover to exchange the distribution of MFs and NN information, as shown in Figure 7. A complete chromosome consists of two substrings: an MF substring and an NN substring. Two points, noted as *a* and *b*, are randomly generated for the MF substrings. Child 1 inherits alleles between the *a* and *b* segments of parent 2. Child 2 inherits alleles between the *a* and *b* segments of parent 1. Complementary portions of the *a* and *b* segments are retained for the other child. To explore the topology and parameters of the NNs, the third cut-point *c* is randomly generated for the NN substring. The produced children exchange the right-hand features after the cut-point from their parents.

#### 3.2.4. Mutation

**Figure 8:**
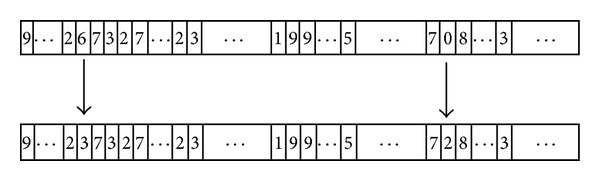
Mutation operation.

The mutation produces spontaneous random changes in various chromosomes, thus protecting against premature loss of important notations. The purpose of mutation is to improve performance by adjusting the value of the summits and widths of MFs, along with interconnections, weights, biases, and activation slopes. It alters one or more genes with a probability (*p* ^ge^), which is smaller than or equal to the mutation rate (*p* ^mu^). Mutation operation compares each gene's *p* ^ge^ with *p* ^mu^. If *p* ^ge^ ≤ *p* ^mu^, then value of the gene is changed to another unrepeated number, as shown in Figure 8.

#### 3.2.5. Selection

The selection process emulates the survival-of-the-fittest mechanism found in nature. It selects a new population with respect to the probability distribution based on fitness for survival. The probability distribution is established using the roulette wheel method [29], constructed as follows:

1. calculate the total fitness for the enlarged sampling space;
2. calculate the selection probability for each chromosome;
3. calculate the cumulative probability for each chromosome.

## 4. Application

### 4.1. Case Study

To validate feasibility of the proposed EFNNs, a real construction company in Taiwan is studied. Establishing in 1956, the company is ISO 9002 certified, with a capitalization of about 11 million USD. Based on Wu's [23] findings, a subcontractor's previous three performances are used to predict its next performance. Historical subcontractor performance records are extracted from Wu [23] and are shown in Table 1. The 34 subcontractor performances shown in the table are real cases based on 14 subcontractors. Of the 34 input patterns, 22 are used to evolve EFNNs, while the unseen 12 test sets are used to validate the generalization of EFNNs.

### 4.2. Web-Based Evolutionary Fuzzy Neural Networks

**Figure 9:**
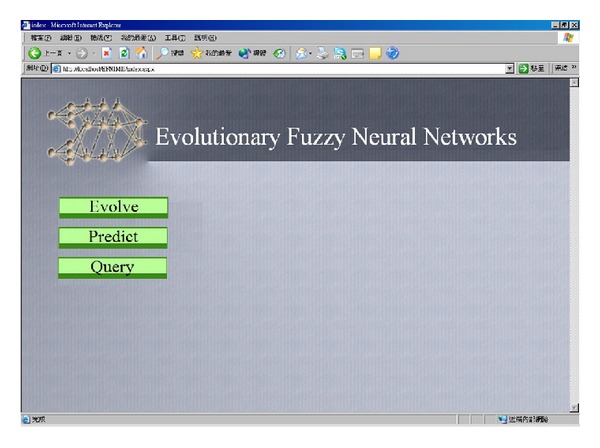
Application main interface.

**Figure 10:**
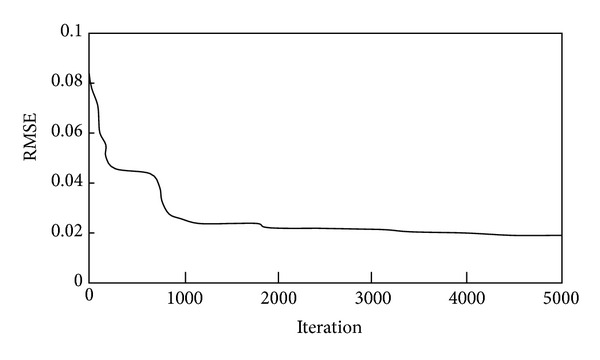
Evolutionary process.

Web-based EFNN software was developed to automate the evolutionary process. The main interface of the web-based system is shown in Figure 9. Three modules are provided in the system. The evolutionary module is used to implement the EFNN evolutionary process. The prediction module can be used to predict subcontractor performance using the network obtained by the evolutionary module. EFNNs fuse GAs, FL, and NNs into an integrated network and thus contain parameters of these three AI techniques, which are summarized in Table 2. The system parameters and database can be manipulated using the query module.  Figure 10 displays the evolutionary process for the case study. The optimum solution is derived at iteration 4947.

### 4.3. Subcontractor Performance Prediction

**Figure 11:**
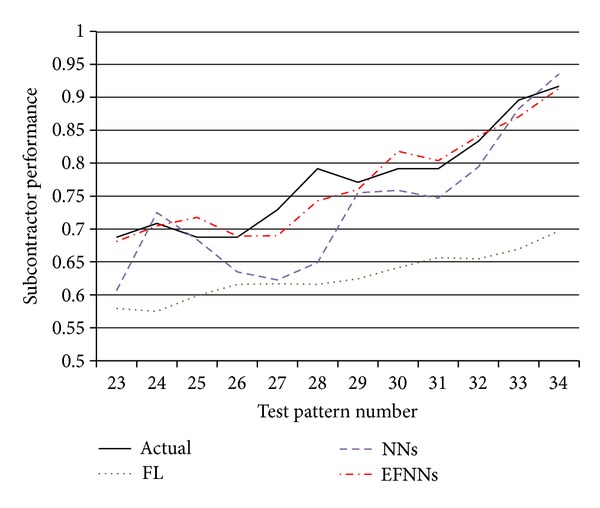
Comparison of prediction results.

Table 3 compares the generalization ability of the proposed method with that of FL and NNs. The test data are not included in the training process. Prediction accuracy is visualized in Figure 11, which shows that subcontractor performance is predicted more accurately using EFNNs than by using FL and NNs. The performance of each method is calculated using the root mean square error (RMSE). In the table, the generalization ability of the EFNNs outperforms that of the NNs, and EFNNs significantly outperform FL. In addition, applying EFNNs for predicting subcontractor performance requires no effort in terms of MF identification, fuzzy rule acquisition, composition operator determination, NN topology configuration, or NN parameter recognition. Thus the proposed approach both improves prediction accuracy and reduces the time required to develop a tool for performance prediction.

## 5. Conclusions

Subcontractor performance is considered an important indicator for general contractors to select subcontractors. To facilitate such decision-making, this study hybridizes NNs, FL, and GAs to develop the EFNNs. Parameters required in NNs and FL are encoded using floating numbers. A multi-cut-point crossover is used to explore the optimum combination of parameters and maintain solution legitimacy. Twelve test cases not used in the evolutionary process are applied to validate the performance of the proposed method. Application results show that the proposed EFNNs outperform NNs and FL in predicting subcontractor performance. Furthermore, users do not need to configure parameters such as membership function distributions, NN parameters and topology, and defuzzification parameters, thus reducing the effort required to develop prediction tools. A web-based application is developed to automate the evolutionary process, thus increasing user convenience. Subcontractor performance is associated with previous outcomes, and predicting future performance depends on identifying this association. The proposed web-based EFNNs system can be used to automatically establish this association, thus enhancing the efficiency of managerial decision-making. The proposed method is one of the first attempts to apply AI methods to predicting subcontractor performance. Future studies may explore different approaches to further enhance prediction accuracy and application convenience.

## Figures and Tables

**Table 1:** Subcontractor performance historical records.

Pattern no.	Performance	Input			Output
		Last 3	Last 2	Last 1	Normalized performance
Input patterns					
1	80	72	76	76	0.8333
2	86	76	76	80	0.8958
3	74	80	76	76	0.7708
4	70	76	76	74	0.7292
5	68	56	62	66	0.7083
6	66	60	66	68	0.6875
7	66	70	72	68	0.6875
8	58	62	66	60	0.6042
9	56	66	60	58	0.5833
10	80	76	74	76	0.8333
11	86	74	76	80	0.8958
12	88	76	80	86	0.9167
13	76	86	80	80	0.7917
14	70	66	68	66	0.7292
15	70	68	66	70	0.7292
16	76	66	70	70	0.7917
17	74	66	70	76	0.7708
18	76	70	76	74	0.7917
19	80	74	76	76	0.8333
20	66	62	58	62	0.6875
21	68	58	62	66	0.7083
22	76	76	74	76	0.7919

Test patterns					
23	66	62	56	60	0.6875
24	68	56	60	66	0.7083
25	66	60	66	68	0.6875
26	66	66	68	66	0.6875
27	70	68	66	66	0.7292
28	76	66	66	70	0.7917
29	74	66	70	76	0.7708
30	76	70	76	74	0.7917
31	76	76	74	76	0.7917
32	80	74	76	76	0.8333
33	86	76	76	80	0.8958
34	88	76	80	86	0.9167

Note: Last 1 denotes the subcontractor's latest performance, and so forth. Normalized performance is divided by 96.

**Table 2:** EFNN parameters.

Technique	Parameter	Value
GAs	Population size	50
	Crossover rate	0.5
	Mutation rate	0.01
	Terminal condition	5000 iterations

FL	Number of fuzzy sets	5
	Defuzzification function	Average output method
	MF shape	Trapezoidal shape

NNs	Connection weight	0.0–1.0
	Bias	−1–0
	Activation function	Heaviside
	Hidden layers	1–6
	Hidden neurons	1–6

**Table 3:** Comparison of prediction results.

Pattern no.	Subcontractor performance	FL predicted performance	NNs predicted performance	EFNNs predicted performance
23	0.6875	0.5793	0.6064	0.6810
24	0.7083	0.5748	0.7247	0.7046
25	0.6875	0.5986	0.6837	0.7175
26	0.6875	0.6158	0.6350	0.6892
27	0.7292	0.6169	0.6225	0.6898
28	0.7917	0.6158	0.6491	0.7428
29	0.7708	0.6245	0.7550	0.7601
30	0.7917	0.6413	0.7588	0.8180
31	0.7917	0.6567	0.7469	0.8038
32	0.8333	0.6546	0.7943	0.8409
33	0.8958	0.6694	0.8826	0.8702
34	0.9167	0.6968	0.9353	0.9132

RMSE		0.1527	0.0624	0.0234

Real performance score is multiplied by 96.
